# Personals

**Published:** 1915-03

**Authors:** 


### Personals.

The wife of Dr. T. A. Moore of Greenwood has been quite sick for some time.

Dr. T. B. Bass has been re-elected superintendent of the State Epileptic Colony at Abilene.

Dr. Winn of Alvin received the appointment of physician to the Clemmens and Retrieve farms.

Dr. Randolph of the Scott & White Sanitarium of Temple is the successor of Dr. H. Kuehne of Walling.

Dr. Hugh S. White was deposed by the commissioners court of El Paso, and Dr. G. N. Thomas was named in his stead.

Dr. W. Van Kanzler, a physician of San Antonio, is interested in the establishment of a city hospital in Corpus Christi.

Dr. Paul Renger has returned to Hallettsville from New Orleans, where he has taken a post-graduate course in medicine and surgery in the Tulane University.

Dr. C. M. Cash of Taylor having resigned as county health officer, his son, Dr. W. Andeve Cash, was appointed by the county commissioners court in his stead.

Dr. H. F. Leach, a prominent physician of Weatherford, and president of the Northwest Texas Medical Society, will sail for Europe under the auspices of the American Red Cross Society at an early date, and will go immediately to the war zone.

